# Are Our Farm Workers in Danger? Genetic Damage in Farmers Exposed to Pesticides

**DOI:** 10.3390/ijerph16030358

**Published:** 2019-01-27

**Authors:** Ana Flavia Marcelino, Catia Cappelli Wachtel, Nédia de Castilhos Ghisi

**Affiliations:** 1 Programa de Pós-Graduação em Agroecossistemas, Universidade Tecnológica Federal do Paraná (UTFPR), Dois Vizinhos 85660-000, Brazil; mrcelinoana@gmail.com (A.F.M.); catia_capelli@hotmail.com (C.C.W.); 2 Programa de Pós-Graduação em Biotecnologia (PPGBIOTEC), Diretoria de Pesquisa e Pós-Graduação da Universidade Tecnológica Federal do Paraná (UTFPR), Dois Vizinhos 85660-000, Brazil

**Keywords:** carcinogens, comet assay, micronucleus test, farmers

## Abstract

Modern agriculture, practiced after the “green revolution” worldwide, aims to maximize production in order to provide food for the growing world population. Thus, farmers are required to modernize their practices through the mechanization of land use and, above all, the use of chemical pesticides to control agricultural pests. However, in addition to combating the target pest, chemical pesticides indirectly affect a wide range of species, including humans, leading to health damage. Among the main problems caused by the use of pesticides is the genotoxicity caused by chronic exposure. The present study aims to verify the occurrence of genetic damage in farmers who are occupationally exposed to agrochemicals compared to people of other professions that do not use toxic substances (control group). The research was conducted with 36 male participants (18 farmers and 18 control group, ages 24–71 for the farmer group and 22–61 for the control group). The comet assay and micronucleus test results revealed a higher rate of genetic damage in the group of farmers than in the control group. A questionnaire answered by the farmers showed that the Personal Protect Equipment (PPE) is used incorrectly or not used. In summary, our results indicate that farmers are exposed to occupational hazards. To mitigate this risk, we conducted awareness campaigns to notify the farmers of the risks and highlight the importance of using PPE correctly. Intensive efforts and training are thus required to build an awareness of safety practices and change the attitudes of farm workers in the hope of preventing harmful environmental and anthropogenic effects.

## 1. Introduction

The use of pesticides in the world has become a common and theoretically necessary practice in modern agriculture with the advent of the so-called “Green Revolution”, which is a wide-scale program to boost worldwide agricultural production [1]. According to Matos [2], increasingly modern and mechanized agricultural practices aiming at higher production and profit are some of the objectives of the Green Revolution. There have been increased production demands for the speedy and effective extermination of pests (undesirable plants, animals or microorganisms) that may compromise production.

The acute effects of pesticides on human health are well known, especially for the most-commonly applied pesticides [3]. However, the chronic effects caused by continuous long-term exposure have not been characterized properly. These effects include carcinogenesis, neurotoxicity, problems in reproductive development and immunological effects. They are also poorly known because the latent effects of some of these chemicals may only become apparent after 18 years of absorption [3].

Genotoxic damage caused by pesticides in DNA has been the target of dozens of studies around the world. It is known that pesticides are substances that are reactive to DNA, which can cause changes in genomic DNA and are therefore recognized as carcinogenic. Exposure to carcinogenic pesticides can lead to damage such as cross-link DNA-DNA and DNA-Protein, chain breakdown and formation of DNA adducts, generating defective cells [4].

Pesticides are absorbed by the human body through the skin and respiratory pathways and, to a lesser extent, orally [5]. The inhalation of pesticides is a concerning issue and an important cause of health problems in humans. Once inside the human body, they can cause acute or chronic intoxication. Acute intoxication is caused by exposure to a particular substance in large doses for a short period and can result in nausea, vomiting, headache, dizziness, disorientation, hyperexcitability, paresthesia, skin and mucous membrane irritation, muscle fasciculation, breathing difficulty, bleeding, seizures, coma and death [5]. Exposure to organophosphates can lead to abnormal sperm, fetal death, birth defects, hormonal changes, DNA damage, and changes in the ovaries and eggs [6].

Chronic intoxication is characterized by late onset after months or years of low or moderate exposure to toxic chemicals or multiple products, leading to irreversible damage, such as paralysis and neoplasia [7]. Mutagenicity is among the worst of the possible damages caused by agrochemicals and deserves consideration due to the irreversible nature of this condition and its long latency period [8]. Studies in the literature report that workers exposed to pesticides are more prone to developing leukemia and prostate, skin, and brain cancers than the general population [9].

In addition to the risk of developing latent conditions like cancer and compromising syndromes, rural workers are susceptible to another type of occupational hazard with irreversible loss: suicide. Studies conducted by Falk et al. [10], in the Rio Grande do Sul state (Brazil), found that suicide is directly related to the use of organophosphate pesticides, and that these pesticides produce neurological sequelae including chronic behavioral effects in the central nervous system, such as difficulty concentrating, memory loss, apathy, irritability, schizophrenia, and depression.

Moreover, the lack of use of Personal Protective Equipment (PPE) can aggravate or increase the rate of genotoxic damage in exposed individuals [11]. According to Koifman and Hatagima [12], regarding genotoxicity, the determination of cytogenetic changes in individuals occupationally exposed to pesticides can be used as a marker of the early biological effects. There is a considerable volume of information about exposure to pesticides, but now, we want to characterize the damage to DNA in farmers. 

Since 2008, Brazil has been the world’s leading pesticide market; and according to the ‘Sustainable Development Indicators Report’ published by the Brazilian Institute of Geography and Statistics (IBGE), the amount of pesticides used per planted area in the country doubled from 2000 to 2012 (3 kg to 7 kg per hectare) [13].

Thus, by using the comet assay and the micronucleus test, this study aimed to assess the damage caused by exposure to substantial amounts of pesticides in the Southwestern Paraná state (Brazil), specifically the risks for farmers who constantly handle and apply these products to their crops. 

## 2. Materials and Methods

This study was previously submitted to the Research Ethics Committee Involving Human Beings of the Federal University of Technology—Paraná (CEP–UTFPR). It was subsequently approved by the Brazilian National Committee on Research Ethics—Comissão Nacional de Ética em Pesquisa (CONEP)—and written informed consent was obtained from all participants before the research began. The Ethical License was approved under protocol No. 1.585.474.

The study was conducted from June to November 2016, in the cities of Santo Antônio do Sudoeste and Pranchita, in the Southwestern Paraná state (Brazil). We contacted 36 men (18 were farmers who used pesticides on their properties and 18 were people who were not exposed to pesticides (control group)). Both groups of study participants were made up of non-smokers. The study participants were all Caucasian. Moreover, in selecting the study participants for both groups, we chose those who reported to not have been affected by any chronic disease in the last six months. A Free and Informed Consent Form was obtained from all men participating in the study. The farmers answered the questionnaire regarding their ages and other questions. The questionnaires were applied only to the farmers because there were specific demographic questions only for this group, such as: “How long have you worked with pesticides? How many pesticide applications do you do per year? What types of pesticides do you use? Do you use PPE when making pesticide applications? Have you ever been poisoned by the use of pesticides? What were the symptoms? How many times have you been through this?” The answers obtained from the questionnaire were analyzed descriptively.

The study participants in the control group reside and work in the same city as the farmers, but live about five kilometers away from the rural area and have no direct contact with pesticides. In these studied regions, there are no applications of pesticides by airplanes. To better characterize these groups, the exposure time and age of the farmers, as well as the occupations and ages of the control group of participants, are presented in the results.

It is important to note that oral epithelium and blood cells were collected on the same day for both groups. In the exposed group (farmers), collections of biological material were done in the morning; in the control group, in the afternoon. The collections took place on a Sunday, aiming to find the largest number of study participants. Participant farmers had not been exposed to pesticides on the day of collection.

### 2.1. Micronucleus Test and Alterations in Nuclear Morphology

The use of buccal epithelium exfoliated cells for the micronucleus assay and the evaluation of nuclear abnormalities is justified because it involves minimally invasive sampling, and with little discomfort to the study participants. It was successfully applied to assess inhalation and exposure to genotoxic agents, the impact of nutrition and lifestyle factors [14]. The protocol used for the micronucleus test was based on Benedetti et al. [15] with modifications. The oral mucosal epithelial cells of the individuals were obtained with a cheek swab, by gently rubbing the inside of the cheeks. The cotton-tip swabs were immersed in a phosphate buffer solution (pH = 6.5) in a 2-mL microtube, refrigerated and transported to the lab. The solution was centrifuged at 1300 rpm for 8 min for sedimentation. The supernatant was removed and the sedimented cells were washed twice with a saline solution (0.9% (w/v) aqueous NaCl). Finally, the sediment was centrifuged with Carnoy’s fixative solution (methanol and acetic acid 3:1) under the same centrifugation conditions. The cells were placed on a clean slide and air dried at room temperature. The slides were stained with 2% Giemsa for 10 min, rinsed in distilled water and air dried.

The slides were analyzed under an optical microscope magnified 400x. A thousand epithelial cells were counted per person. Every considered cell should have intact nuclear membranes. The formation of micronuclei and abnormal changes in the morphology of the nucleus was evaluated and classified in micronucleus, binucleated, bridge, and nuclear buds following Fenech [15,16]. All the considered alterations should have the same staining characteristics of the main nuclei.

Micronuclei were characterized as a supernumerary nuclear structure within the cytoplasm of the cell; round, almond, or ovoid shaped; having a diameter between 1/16th and 1/3rd of the mean diameter of the main nuclei and not connected to it; having staining characteristics consistent with the chromatin of the main nucleus; and having a non-refractory image. Binucleated (BN) cells have two nuclei within the same cytoplasmic boundary, should be approximately equal in size, staining pattern and staining intensity. The two nuclei within a BN cell may be attached by a fine nucleoplasmic bridge which is no wider than 1/4th of the nuclear diameter. Bridge: the width of a nucleoplasmic bridge does not exceed 1/4th of the diameter of the nuclei within the cell. Nuclear buds: extruded nuclear material that appears like a micronucleus with a narrow nucleoplasmic connection to the main nucleus or nuclear blebs that have an obvious nucleoplasmic connection with the main nucleus [17].

### 2.2. Comet Assay

The protocol used to perform the test was based on the methodology described by Singh et al. (1988) with some modifications by Tice et al. [18]. A total of 4 mL of peripheral blood was collected from each individual. The samples were collected and conditioned in tubes with anticoagulant (EDTA) and wrapped in tinfoil to protect them from light exposure. 

After collection, the samples were refrigerated at 4 °C in the Laboratory of Molecular Biology of the UTFPR (University campus Dois Vizinhos). The samples remained refrigerated for 24 h for the complete sedimentation of the red blood cells and separation of the white blood cells and plasma. The plasma was removed with a Pasteur pipette, and 5-µL samples were collected from the portion of leukocytes. These samples were embedded in 75 µL of 0.75% low-melting-point agarose at 37 °C (heated in a water bath) and spread on pre-covered microscopy slides with 1.4% normal agarose. The slides were then covered with a coverslip and refrigerated at 4 °C until solidification (protected from light).

After solidification, the coverslips were removed and the slides were stored in glass vats protected from the light and with ice-cold lysis solution (2.5 M NaCl, 100 mM EDTA and 10 mM Tris, pH 10.0 with 1% (v/v) Triton X-100, and 10% (v/v) dimethyl sulphoxide). The slides remained in a lysis solution at 4 °C until electrophoresis.

The slides were subsequently placed in a horizontal electrophoresis cube, covered with an alkaline electrophoresis buffer (300 mM NaOH and 1 mM EDTA, pH > 13), and remained for 30 min for DNA unraveling, protected from the light. Electrophoresis was performed for 25 min at 25 V and 300 mA. The electrophoresis system was surrounded by ice to keep the temperature at 4 °C and protected from the light.

After electrophoresis, the samples were neutralized with the Tris buffer (0.4 M Tris, pH 7.5). This step was repeated three times, with the renewal of the buffer every 5 min. The slides were rinsed twice in distilled water and dried overnight at room temperature. 

The slides were then fixed in absolute ethanol for 10 min and incubated to dry at 37 °C for an hour and a half. They were hydrated for 5 min in distilled water and stored in slide boxes. For the purpose of analysis, the slides were stained with ethidium bromide (0.02 mg∙mL^−1^), protected from the light, and observed in an epifluorescence microscope.

A total of 100 nucleoids were analyzed for each person using the visual classification based on the migration of DNA fragments, according to the following classes: 0 (no apparent damage), 1 (little damage), 2 (moderate damage) 3 (extensive damage), 4 (maximum damage, apoptosis). After obtaining the values that qualified the damage in lymphocytes (damages 0, 1, 2, 3, and 4), the data were tabulated and the damage scores for each individual were calculated using the following equation: SCORE = ∑ (0 × number of cells with damage 0) + (1 × number of cells with damage 1) + (2 × number of cells with damage 2) + (3 × number of cells with damage 3) + (4 × number of cells with damage 4).

### 2.3. Correlation Damage Rate vs. Age

The ages of farmers were computed to check the relationship between the age and the damage rate of everyone, both for the micronucleus test and comet assay. The damage rate and age data were compared using a 2D scatter plot with simple linear regression for each variable to obtain a p-value and regression equation and to determine if there is a correlation between increasing age and increasing rate of damage.

### 2.4. Statistical Analysis 

The comet assay scores, the data obtained in the micronucleus test, and the nuclear morphological abnormalities were submitted to assumption tests to verify normality and homoscedasticity. The data were then analyzed using a Student’s *t*-test (R software) [19]. The correlation between ages vs. damage rates, both for the comet assay and the micronucleus test, was calculated using Statistica (data analysis software system developed by StatSoft, 2007) version 8.0 [20]. A value of *p* < 0.05 was considered statistically significant.

## 3. Results

After the questionnaire was presented to the farmers, the use of PPE was minimal. When asked if they used PPE, the answers showed that 55.6% of farmers did not use full PPE and 44.4% used it incorrectly, i.e., only some items such as boots, pants and long-sleeved shirts were used, leaving aside the mask, visor and protective cap, damaging the protection provided by PPE. When questioned about which PPE items they used, none of the volunteers replied that they wore all protective items: Arabic cap (which protects neck and shoulders), visor, respirator, waistcoat and water repellent trousers, gloves, apron and waterproof boots. Regarding the reasons why they did not use full PPE, 90% of the volunteers stated that it was uncomfortable and hot.

### 3.1. Micronucleus Test and Alterations in Nuclear Morphology

**Figure 1:**
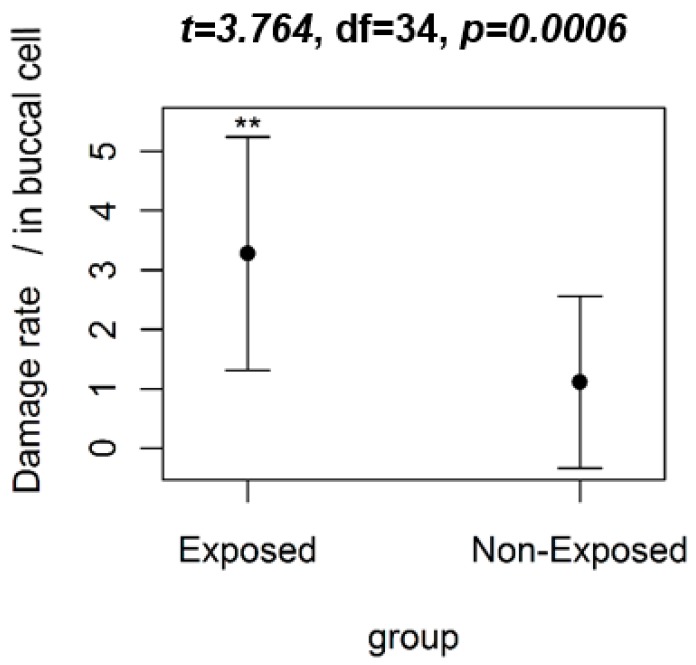
The comparative graph of the damage rates (mean ± SD) observed in buccal cells using the micronucleus test and nuclear abnormalities with the group of farmers (exposed) and the control group (unexposed). The result of the Student’s *t*-test (*t*) is shown in the title. Note: ** significant difference (*p* < 0.05), df = degrees of freedom.

The statistical analysis revealed that the saliva samples collected from the group of farmers (exposed) had an average damage rate of 3.28 alterations for every 1000 cells. The control group (unexposed) had an average of 1.11 morphological changes per 1000 cells. The values of the *t*-test were: *t* = 3.76; degrees of freedom = 34; *p* < 0.05 in the group comparison (Figure 1). Therefore, there is a significant difference between the averages and the farmers had the highest damage values in the micronucleus test.

### 3.2. Comet Assay

**Figure 2:**
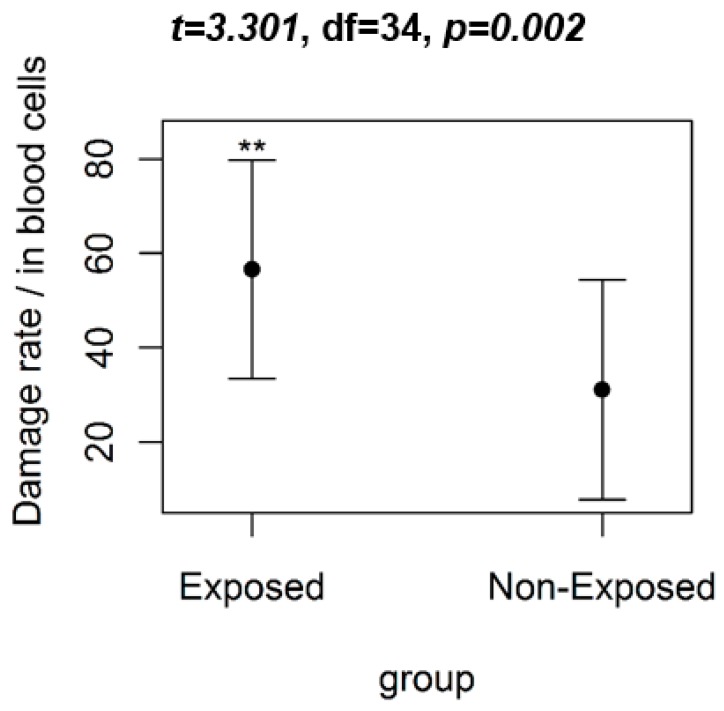
The comparative graph of the damage rates (mean ± SD) observed in the blood cells using the comet assay with the group of farmers (exposed) and the control group (unexposed). The result of the Student’s *t*-test (*t*) is shown in the title. Note: ** significant difference (*p* < 0.05), df = degrees of freedom.

The comet assay revealed that the average damage to the genetic material of the group exposed to pesticides was significantly higher than the average of the control group. The values obtained in the *t*-test were: *t* = 3.301; degrees of freedom = 34; *p* < 0.05. As noted in Figure 2, the group formed by farmers (exposed) had a greater rate of damage to lymphocytic DNA than the control group (unexposed).

### 3.3. Correlation Damage Rate vs. Age

**Figure 3:**
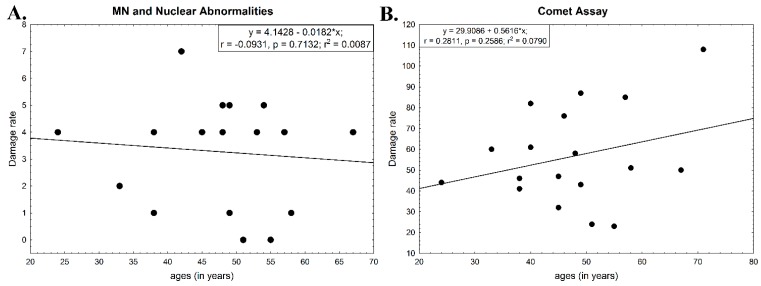
The 2D scatter plots with simple linear regression, showing the correlation between ages of farm workers and damage rate, assessed by (**A**) micronucleus (MN) test and nuclear abnormalities; and (**B**) comet assay.

After comparing the ages of the farmers with the damage rates, it was observed that no significant relationship was found between the age of the study participants and the observed damage rate, as shown in Figure 3. The statistical test using the ages of the men of each test and the corresponding damage rate resulted in *p* > 0.05 for the micronucleus test and for the comet assay. These *p*-values were not significant and, therefore, it was not possible to predict the dependent variable value as a function of the independent variable (damage rate vs. age).

### 3.4. Feedback to Study Participants

Once the tests were completed, the study participants received feedback. This feedback was offered to those who, at the time of collecting the biological material used in the research and completion of the Informed Consent Term (TCLE), opted to know the result. The results were given individually through printed material, so they were informed that this result did not demonstrate any disease but rather damages that, when accumulated in the long run, could have negative impacts on their health. In addition, the researchers offered a lecture to present alternatives to reduce pesticide exposure and, hence, the rate of genotoxic damage, the importance of using PPE in handling any amount of pesticides in addition to the maintenance and cleaning of PPE and the suitable forms of use. During the lecture, the results obtained in the comparison between the farmers’ group and the control group was exposed so that they could understand the importance of the research.

## 4. Discussion

In this study, the micronucleus test detected the increased occurrence of morphological changes in the nucleus of cells of individuals constantly exposed to pesticides. The micronuclei are formed in cells that, following completion of the cell cycle, leave fragments of chromosomes or full chromosome (that are not integrated to the nucleus of the daughter cell) behind in the anaphase of cell division [21]. According to Giri et al. [22], organophosphate pesticides can potentially induce the formation of micronuclei.

Benedetti et al. [15], also compared farm workers who were occupationally exposed to pesticides and people from other professions (who had no contact with toxic substances) in the Rio Grande do Sul state (Brazil). The result of the study by Benedetti corroborates with our findings, where there was a greater presence of micronuclei and a higher damage rate in the comet assay in the cells of farmers.

The comet assay and the micronucleus test revealed that the group of farmers had a higher risk of breaking double-stranded DNA [23]. The Comet assay is a test that is widely used in several areas, including human biomonitoring and genotoxicity, and it efficiently assesses the damage to and repair of DNA in different types of cells in response to a range of DNA-damaging agents [24].

Some studies obtained similar results, such as Garaj-Vrhovac and Zeljezic [25], who compared the DNA damage rate of workers in pesticide production exposed to different formulations with the rate of people who had no contact with these toxic agents. These authors also found a significant difference between the groups. The test was repeated with workers who had not been exposed to pesticides for six months. They continued to have significant genotoxic damage, leading to the conclusion that, in addition to the damage, pesticides can cause changes in the mechanisms that repair mutations [26].

Similar to the micronucleus test, Benedetti et al. [15] observed a significant difference in the number of lesions in the DNA of farmers subjected to the comet assay. A similar result was also found by Kvitko et al. [27], corroborating with our results for the comet assay. These studies were conducted with study participants who also exposed themselves to different formulations of pesticides in their work. Exposure to the chemical mixture of different formulations can lead to damage like cross-link DNA-DNA and DNA-protein, rupture of the two chains of DNA, and the formation of DNA adducts [25].

The comet assay has been used for the occupational biomonitoring of workers. Valverde and Rojas [28] stress the importance of analyzing long-term genotoxic effects to precisely establish the risks of developing cancer, for example. According to Maluf and Riegel [29], lymphocytes, which were the cells used in the comet assay, can reflect the general state of an organism as they circulate through the entire human body. Arshad et al. [6] showed a strong correlation between DNA damage and pesticide concentration in the blood.

This situation can be worsened by continual exposure to pesticides without proper protection because the persistent cytological damage they cause can lead to a higher level of cytogenetic changes. This was reported by the Bull et al. [11] study, where it was observed that the lack of use of PPE in the application of pesticides directly reflected the increase of cytological damage. The problem is also related to the conditions of the PPE since it is not always replaced as needed. Cytologically, if any DNA damage affects the tumor suppressor genes or oncogenes, the damaged cell can differentiate and proliferate uncontrollably, leading to the formation of tumors [30]. Beyersmann and Hartwig [31] related metallic pesticides with inductors of oxidative damage in cells, generating a chain of events that can inhibit DNA damage repair, the accumulation of mutations, and the deregulation of cell proliferation or the inactivation of tumor suppressor genes.

Our study found no clear correlation between the age of the study participants and the damage rate, either by the micronucleus test or by the comet assay, however, studies performed by other authors such as the meta-analysis of Merhi et al. [9] concluded that a long period of exposure to pesticides (more than 10 years) may increase the risk of hematopoietic tissue tumors and non-Hodgkin’s lymphoma. Ferraz et al. [32], with healthy subjects aged in two groups (19–29 years old × over 60 years old), show that the frequency of micronuclei and nuclear degenerative changes was significantly higher among the older group. The same authors concluded that adopting a healthy lifestyle, avoiding the use of pesticides or at least reducing their exposure could minimize the effects of aging, reducing the risk of developing degenerative diseases.

When the farmers were asked about the number of times they had applied pesticides to their properties during the year, their responses were surprisingly high, reaching the minimum number of 10 applications per crop (soybean, wheat, or corn). These applications include desiccation when all the existing vegetation cover (including weeds) is eliminated, which reportedly occurs twice before planting. In addition to the desiccation, the farmers reported to applying fungicide up to four times per crop and that at each pest attack, whether by insects, fungus or weeds, they would administer pesticides.

When asked about which pesticides they used, in the class of herbicides they mentioned glyphosate and 2,4-D (2,4-dichlorophenoxyacetic acid). The use of 2,4-D has been expanded in the Paraná state according to data provided by the Agency of Agricultural Defense of Paraná [33]. Among the fungicides, they cited the active ingredients azoxystrobin (pyrazole), cyproconazole (-triazol), pyraclostrobin (carbamate) and epoxiconazol (-triazol). In the class of insecticides, the farmers mentioned profenofos, lufenuron (organophosphates), and tiametoxam (pyrethroid). According to data provided by ADAPAR, the pesticides with the highest total sales in the state in 2015 were glyphosate and its equivalent acids, with about 13.05%, while another herbicide mentioned by the study participants, 2.4-D, had 2.02% of the total sales. Azoxystrobin and cyproconazole also accounted for more than 1% of the total sales. The other active ingredients mentioned by the farmers totaled 1% of the total sales each and are included on a list with another 365 compounds that are sold in the Paraná state [33]. According to ADAPAR, in 2015, 47.34% of the pesticides sold were for soy, 16.66% for corn, and 10.31% for wheat [33].

Monroy et al. [34] used the comet assay to study the effects of glyphosate on human cells in vitro. DNA damage was observed after treatment with this pesticide, which is the most widely sold in the Paraná state. A similar result was found by Mañas et al. [35], showing that glyphosate causes genotoxic changes in human liver cells and increases the number of micronuclei in mice.

Similar to this study, Paiva [36] performed biomonitoring on farmers from two communities in the Ceará state (Brazil) using the comet assay. Data collected by this author show that farmers in the region also use the pesticides azoxystrobin and cyproconazole. The comet assay also revealed a significant result between the damage found in the group of farmers and the control group, with a high damage rate among the farmers.

Concern about the genetic damage caused by pesticides is worldwide, as their use has become extremely diffuse in all agricultural crops. Even children living in extremely agricultural areas end up being affected. Studies have identified significant amounts of genotoxic damage in children exposed to multiple formulations of pesticides in Poland and also in Mexico [37,38]. In addition, in Greece, oxidative damage was also found in the DNA of pesticide sprayers [39].

The present study demonstrated that farm workers are constantly exposed to cytotoxic and mutagenic substances, as shown in the micronucleus test and comet assay. Thus, the need for biomonitoring studies to assess and ensure good working conditions for farmers and to provide greater awareness regarding the use of PPE is remarkable. Consequently, in addition to researching genetic damage, this work also provided feedback to the study participants through an awareness lecture for the participants of this project and their families.

We also stress the need to disseminate research in this area and the use of less toxic pesticides or alternative methods of pest control in agriculture.

## 5. Conclusions

In this study, the rate of genetic damage in farmers who are occupationally exposed to pesticides was found to be significantly greater than in the unexposed group that does not work with toxic substances.

Moreover, the questionnaires showed that the farmers do not correctly use the PPE recommended for spraying pesticides, which may have contributed to the high genotoxic damage rate.

No significant relationship was found between the damage rate and the age of the study participants.

With the awareness action, the farmers were informed of the risks they were taking with the exposure to pesticides. However, they seemed unwilling to change their work practices, such as the full and indispensable use of PPE when dealing with any amount of pesticide.
